# Differentiation Potential of Pancreatic Fibroblastoid Cells/Stellate Cells: Effects of Peroxisome Proliferator-Activated Receptor Gamma Ligands

**DOI:** 10.1155/2011/816791

**Published:** 2011-10-04

**Authors:** M.-L. Kruse, S. Hopf-Jensen, C. Timke, B. Agricola, G. Sparmann, A. Schmid, B. Sipos, A. Arlt, H. Schäfer

**Affiliations:** ^1^Laboratory for Molecular Gastroenterology and Hepatology, Department for General Internal Medicine, University Hospital Schleswig-Holstein, Campus Kiel, Arnold-Heller-Str. 3 (Building 6), 24105 Kiel, Germany; ^2^Klinik fuer Diagnostische und Interventionelle Radiologie/Neuroradiologie, Diakonissenanstalt zu Flensburg, Knuthstr. 1, 24939 Flensburg, Germany; ^3^Department of Pediatrics, University Hospital Schleswig-Holstein, Campus Kiel, Arnold-Heller-Str. 3 (Building 9), 24105 Kiel, Germany; ^4^Institute for Clinical Cytobiology and Cytopathology, University of Marburg, Robert-Koch-Str. 6, 35037 Marburg, Germany; ^5^Division of Gastroenterology, Department of Internal Medicine, University of Rostock, Ernst-Heydemann-Straße 6, 18057 Rostock, Germany; ^6^Department for General and Thoracic Surgery, University Hospital Schleswig-Holstein, Campus Kiel, Arnold-Heller-Str. 3 (Building 18), 24105 Kiel, Germany; ^7^DRK Krankenhaus Moelln-Ratzeburg, Röpersberg 2, 23909 Ratzeburg, Germany; ^8^Institute for Pathology, University Hospital Schleswig-Holstein, Campus Kiel, Arnold-Heller-Str. 3 (Building 14), 24105 Kiel, Germany; ^9^Department of General Pathology, University of Tuebingen, Liebermeisterstraße 8, 72076 Tuebingen, Germany

## Abstract

Pancreatic stellate cells have been investigated mostly for their activation process, supposed to support the development of pancreatic disease. Few studies have been presented on reversal of the activation process in vitro. Thiazolidinediones (TZDs) have been used as antidiabetics and have now been reported to exert antifibrotic activity. We tested effects of natural and synthetic ligands of peroxisome proliferator-activated receptor gamma (PPAR*γ*) on human pancreatic fibroblastoid cells (hPFCs) in search for specificity of action. Ciglitazone, as a prototype of TZDs, was shown to have reversible growth inhibitory effects on human pancreatic fibroblastoid cells/stellate cells. Cells treated with ciglitazone for three days showed enhanced lipid content and induction of proteins involved in lipid metabolism. Collagen synthesis was reduced in hPFC. Interaction of PPAR*γ* with DNA binding sites upon ligand binding was shown by gel shift analysis. These findings point toward a potential for adipocyte differentiation in human pancreatic fibroblastoid cells.

## 1. Introduction

Pancreatic stellate cells (PSCs) are interstitial cells of the pancreas [1], which in vitro are rapidly activated and develop the phenotype of myofibroblasts, characterised by enhanced expression of alpha smooth muscle actin (*α*SMA). This development is associated with a loss of lipid storage [2]. Activation of PSC and differentiation into myofibroblasts in vitro is assumed to be the process underlying activation of PSC in vivo, leading to enhanced production of extracellular matrix, and ultimately, towards fibrosis. Pancreatic fibrosis is found in chronic pancreatitis (CP), or as a desmoplastic reaction in pancreatic ductal adenocarcinoma (PDAC). In rats, deposition and degradation of extracellular matrix is a highly balanced process that occurs during regeneration of the pancreas after induction of acute pancreatitis [3]. Repeated injury most likely leads to impairment of regulatory mechanisms and thus shifts the balanced process towards matrix deposition and detection of *α*SMA-positive interstitial cells or myofibroblasts [4]. A similar deregulation could be induced by persistent exposure to toxic agents, such as organic zinc compounds [5]. Pancreatic fibroblasts, or stellate cells, are major producers of extracellular matrix, and thus activation and deregulation of these cells is most likely the key event in formation of fibrotic deposits. Addressing the process of activation appears to be a means towards the treatment of fibrosis. 

Thiazolidinediones, antidiabetic drugs, and synthetic ligands of peroxisome proliferator-activated receptor gamma (PPAR*γ*) have been shown to inhibit the activation of PSC in vitro [6], and in animal models, PPARgamma ligands have been shown to ameliorate the development of chronic pancreatitis [7, 8]. PPAR*γ* is a nuclear receptor that dimerises with retinoid-X-receptor to bind to DNA of target genes. PPAR*γ* appears to be a central regulator in lipid metabolism and adipocyte differentiation [9]. While PPAR*γ* ligands have been shown to inhibit culture activation of PSC and maintain a more quiescent state in freshly isolated cells [6], overexpression of PPAR*γ* itself in immortalized PSCs inhibits proliferation and reduces collagen synthesis [10]. In this study, we describe the ultrastructural morphology of human pancreatic fibroblastoid cells isolated by outgrowth from human pancreatic tissue samples in comparison to rat pancreatic stellate cells. The effect of PPAR*γ* ligands on human pancreatic fibroblastoid cells in vitro and a potential for adipogenic differentiation were investigated.

## 2. Materials and Methods

### 2.1. Materials

All chemicals were of highest analytical purity purchased from Sigma, Deisenhofen, Biomol Hamburg, or Merck, Darmstadt, Germany.

### 2.2. Animals, Tissue, and Cell Culture

All animal work was carried out according to the procedural and ethical guidelines of the local Animal Care and Use Committee. PSCs were isolated from female BN/LEW rats, as described before by isopycnic density centrifugation [11], and were allowed to adhere to culture dishes for 3 days prior to further processing. Human pancreatic tissue samples were derived from different patients who gave informed consent according to institutional ethical procedures. For explantation of human pancreatic tissue, culture dishes were either coated with Matrigel (Becton Dickinson) at a concentration of 0.5 mg/mL, or rat tail collagen, or left untreated. Pancreatic tissue was cut into small cubes of roughly 2 mm^3^ and placed into droplets of heterologous human blood plasma containing EGTA. Coagulation of plasma was started by adding Thromborel solution (Dade-Behring, Marburg, Germany) and calcium solution (10 mM), keeping the tissue in a plasma clot in place. Finally, DMEM (Dulbecco's modified Eagle's medium) supplemented with 10% heat-inactivated horse serum, 20% FBS (fetal bovine serum (Biochrom, Berlin, Germany)), 10 mM HEPES, and antibiotics (Amphotericin B, 10 *μ*g/mL streptomycin, 10 U/mL penicillin, and 5 *μ*g/mL gentamicin) was added, and tissue explants were incubated at 37°C in a humid atmosphere under 5% CO_2_. Medium was changed every three days. Outgrowing cells were documented using a Zeiss Axiovert microscope (Carl Zeis Jena, Germany). For isolation and passage of outgrowing cells, the plasma clots with remaining tissue were removed, and the cell layer was trypsinised for 10 min at 37°C. Cells were plated with ECM, RTC, or on untreated plates in medium containing 10% FBS. Transformed HPF-T cells were generated by transfection with a plasmid containing SV40 large T antigen. Cells were maintained in culture as described above, using DMEM supplemented with 10% FBS and 100 *μ*g/mL geneticin. Expression of large T antigen increased lifetime of the cells but did not stably immortalise them. Spontaneously immortalized rat pancreatic fibroblastoid cells were cultured as a stable cell line called PFC1. The cells were derived from rat pancreas after induction of pancreatitis with cerulein [12]. Cells were kept in DMEM supplemented with 10% FBS as described above.

### 2.3. Cell Proliferation Test

For investigation of proliferation as a direct measure of viability, cells were plated onto 96-well plates at a density of 2500 cells per well. After 24 hours of culture, cells were washed with PBS and switched to serum-free medium for another 24 hours. Subsequently, cells were treated with the indicated agents for the indicated times, and 4 h before the end of treatment, MTS reagent (Promega, Madison, Wis) was added, and colour development was monitored every 60 min up to 4 h using a Dynatech MR 5000 plate reader. All measurements were carried out in triplicate in at least 3 different experiments (*n* ≥ 3). Significance was determined by student's *t*-test.

### 2.4. Oil Red Staining

For morphological investigation, cells were plated onto glass coverslips with or without extracellular matrix compounds in 12-well plates (Costar, Acton, Mass). Samples were fixed in 2.5% paraformaldehyde (PFA) in PBS for 20 min at room temperature (RT). Oil red staining was carried out on PFA-fixed material, for 10 min in a saturated solution of oil red (Polysciences, Warrington, Pa) in ethanol/acetone, washed in distilled water, counterstained with hematoxylin (Vector Laboratories, Burlingame, Calif), and mounted in glycerol gelatine.

### 2.5. Fluorescence-Activated Cell Sorting (FACS)

For flow cytometry analysis, cells were plated at a density of 200.000 cells per well onto 6-well plates and grown overnight. Cells were then incubated with serum-free medium for 24 h and subsequently treated as indicated. At the end of incubation, cells were washed with PBS containing 0.1% sodium azide, and all supernatants were collected; subsequently cells were detached by incubation with trypsin solution for 10 min, neutralised by addition of growth medium, and collected by centrifugation. After another wash in PBS containing azide, cells were treated for analysis. Lipid content was determined by nile red staining. Cells resuspended in 1 mL of PBS were incubated for 5 min with nile red solubilised in DMSO at a concentration of 1 *μ*g/mL in the dark. Analysis was carried out using a Galaxy Analyser (DAKO, Denmark) and FlowMax software (DAKO, Denmark) at 488 nm wavelength for excitation with detection filters for FITC and PE for nile red [13]. For cell cycle analysis, cells resuspended in 0.5 mL PBS-EDTA were fixed by addition of an equal volume of ethanol and incubated for 30 min at room temperature. After washing with PBS-EDTA, cells were treated for 30 min with RNase A at a concentration of 2 *μ*g/mL for 30 min. After another wash with PBS-EDTA, propidium iodide (10 mg/mL) was added, and cells were incubated in the dark for 15 min. Subsequently, cells were analysed as described above (*n* ≥ 3). Significance was determined by student's *t*-test.

### 2.6. RNA Isolation and PCR (Polymerase Chain Reaction) Analysis

RNA isolation was carried out using TriFast reagent (PeqLab, Germany), according to the manufacturer's protocol. RNA was solubilised in RNase-free water with RNase inhibitor added and stored at −80°C. Reverse transcription of 1 *μ*g of RNA was carried out with oligo-dT priming and SuperScript 2 reverse transcriptase (Invitrogen) according to the manufacturer's protocol, and the resulting cDNA was stored at −20°C. For amplification, 1 *μ*L of cDNA was used. PCR was carried out with HotStar Gold PCR mix (Eurogentech, Belgium), using the following primer pairs for human RNA: GAPDH (5′-TGAAGGTCGGAGTCAACGGATTTGGT-3′, 5′-CATGTGGGCCATGAGGTC-CA-CCAC-3′, PPAR*γ* (5′-AACTGCGGGGAAACTTGGGAGATTCTCC-3′, 5′-AATAATAA-GGTGGAGATGCAG-GCTCC-3′), LPL (5′-CTTGGAGATGTGGACCGAC-3′, 5′-GTGCCATACAGAGAA-ATCTC-3′), FAPB (5′-TTGCTACCAGGCAGGTGGCC-3′, 5′-CCAGTGTGGTCTCTT-GCCCG-3′). Products were resolved on 8% polyacrylamide-TBE gels, stained with ethidium bromide, and documented with Polaroid film (Kodak).

### 2.7. Procollagen I Propeptide EIA

For analysis of collagen synthesis, cells were plated in 6-well plates and incubated in 1 mL of serum-free medium containing either ciglitazone at a concentration of 3.3 *μ*M, or 15Δ-prostaglandin J_2_ at 2.5 ng/mL. Incubation was carried out for 24–72 h. Supernatants were collected, and cells were harvested for determination of protein content. Medium was changed every 24 h, if applicable. Determination of collagen synthesis (*n* ≥ 3) was carried out using a procollagen type I C-peptide (PIP) EIA kit according to the manufacturer's protocol (Takara Bio Europe S.A.S., France). The assay is specific for human procollagen I propeptide released into the cellular supernatant upon collagen assembly, thus it represents a direct measure of collagen synthesis. Significance was determined by student's *t*-test.

### 2.8. Electrophoretic Mobility Shift Assay (EMSA)

Isolation of nuclear extracts was carried out according to Sikora et al. [14], labelling of probes, incubation, and gel analysis was done as described before [15]. Nucleotide probes were consensus sequences for RXR and PPAR*γ* consensus sequences from Santa Cruz (San Jose, Calif) and a sequence of rat acyl-coenzyme A oxidase (5′-gggaacgtgacctttgtcctggtccc-3′) containing PPAR-response elements.

### 2.9. Electron Microscopy

Cells were fixed in situ with either 3% glutaraldehyde (GA) in cacodylate buffer at pH 7.2 containing 0.1% malachite green [16] or with 2.5% GA and 2.5% PFA in cacodylate buffer for at least 30 min at 4°C. Further processing was carried out essentially as described before [17]. Analysis was carried out using EM109 and EM 902 (Zeiss, Oberkochen, Germany).

## 3. Results

**Figure 1:**
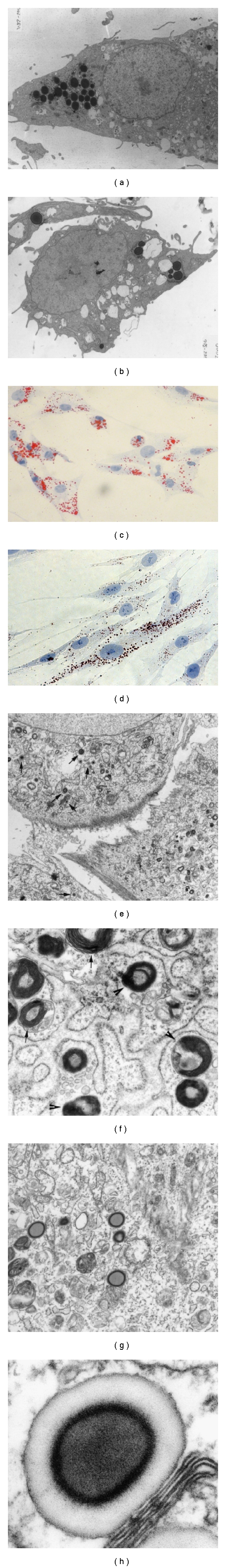
Morphological analysis and comparison of PSC and hPFC. (a) Pancreatic stellate cells after 3 days of adherence fixed with glutaraldehyde/malachite green to preserve lipid droplets. (original magnification: 3000x). (b) PSC in standard fixation showing layered lipid droplets (original magnification: 3000x). (c) Oil red staining of PSC after 3 d in culture (original magnification: 200x). (d) Oil red staining of human pancreatic fibroblastoid cells (hPFCs) after 3 passages (original magnification: 200x). (e)–(h) Electron microscopy analysis of hPFC in standard fixation after 3 passages. (e) Section of three adjacent cells showing dense cytoskeletal structures at sites of adhesion. Throughout the cytoplasm, multilamellar vesicles and round layered entities (arrows), can be seen (original magnification: 3000x). (f) Multilamellar vesicles at higher magnification (20000x) show different stages of membrane degradation. While some structures show stacked lipid bilayers (arrow), others contain electron-dense “molten” membrane in addition to fuzzy, lighter material (arrowheads). (g) Picture shows layered lipid entities next to multivesicular lysosomal/autosomal structures (original magnification 7000x). (h) High magnification view (85000x) of layered entity reveals these structures as nonmembrane-bounded lipid droplet.

Human pancreatic fibroblastoid cells (hPFCs) were isolated by outgrowth from human pancreas tissue explanted into tissue culture plates similar to rat PFCs, as described before [12]. For comparison, pancreatic stellate cells (PSCs) were isolated from rat pancreatic tissue by differential centrifugation after tissue digest, as described before [11]. Rat PSCs were fixed after 3 days of adherence using glutaraldehyde/malachite green (GAM) for lipid preservation, as depicted in Figure 1(a), and a standard fixation, seen in Figure 1(b). Lipid droplets were prominent in PSCs after 3 days in culture. They appeared as mostly round dark, electron-dense structures without a membrane boundary and mostly unstructured in GAM fixation (Figure 1(a)). Conventional fixation revealed a more structured appearance, with a darker outer rim, but still no membrane boundary (Figure 1(b)). Alongside the lipid structures, large, mostly empty vacuoles were seen throughout the cytoplasm. Oil red staining of PSCs after 3 days in culture, as depicted in Figure 1(c), showed large, bright red irregular-shaped entities throughout the cytoplasm of the cells. Oil red staining of human pancreatic fibroblastoid cells (hPFCs) after three passages revealed a quite different picture, as seen in Figure 1(d), and the staining pattern shows small dots, which are more abundant in some cells, while other cells are almost devoid of oil red-positive particles. Ultrastructural analysis of hPFC did not show prominent lipid droplets, but multiple multilamellar vesicles and small structured nonmembrane bounded entities (arrows in Figure 1(e)). Higher magnification showed stacks of lipid bilayers within membrane compartments (arrows in Figure 1(f)) and structures with reduced, fuzzy, or “molten-” looking lipid layers (arrowheads in Figure 1(f)). Right next to regular multivesicular structures, nonmembrane-bounded homogenous lipid structures could be seen (Figure 1(g)), which gave a similar appearance with darkened outer rim, as seen in rat PSCs. High-resolution analysis as seen in Figure 1(h) revealed the amorphous nature of a lipid structure, made up of layers of different electron density, without a defining outer lipid bilayer, which is a characteristic of lipid droplets.

**Figure 2:**
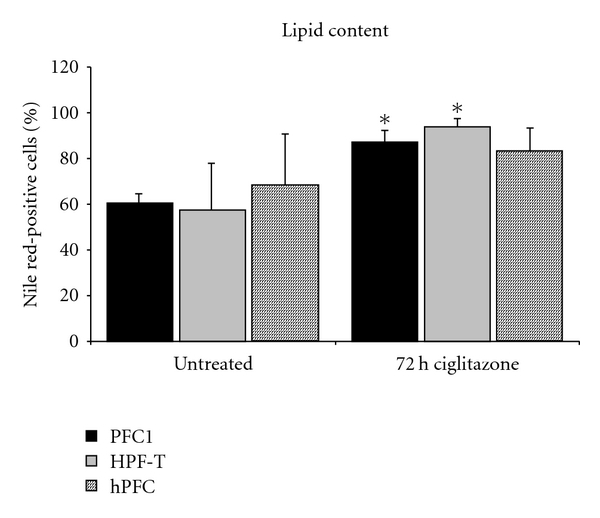
Lipid content evaluated by nile red binding. The figure shows the effect of 72 h of ciglitazone treatment on human pancreatic fibroblastoid cells (hPFCs, hatched columns), transformed hPFCs (HPF-T grey columns), and rat PFC1 cells (black columns) in comparison. Nile red binding to cells was evaluated by flow cytometry and calculated as % of nile red-positive cells. Ciglitazone-induced nile red binding in all cells tested, and hPFC showed a high variation of lipid content in untreated cells with the mean higher than any other cell line tested, but with *P* = 0.053, the rise in lipid content was not significant. (Depicted are means plus standard deviation, * significant versus untreated, *n* ≥ 3.)

As seen in oil red staining and at ultrastructural level, lipid droplets were not uniformly distributed in every cell. In order to quantitate a large number of cells and investigate whether lipid storage was a generalised feature, not limited to single individual cells, we used binding of nile red. Nile red is a dye with high affinity for lipids and allows for flow cytometry evaluation. Isolates of hPFC from individual human pancreatic tissues are inhomogeneous in appearance and often limited in cell numbers, due to a limited lifetime. We employed a stable cell line of rat pancreatic fibroblastoid cells, named PFC1, and transformed human pancreatic fibroblastoid cells, named HPF-T (see Section 2) for comparison. Cells were treated with ciglitazone, a member of the thiazolidinedione family of PPAR*γ* ligands to test for its possible influence on lipid storage.  Figure 2(a) shows the results of 72 h treatment with 3.3 *μ*M ciglitazone. PFC1 cells showed a mean content of nile red-positive cells of about 60% which rose significantly by 27% after 3 days of ciglitazone treatment. The transformed human cells, HPF-T, showed a significant increase of lipid content by 36%. Human PFCs revealed a higher amount of nile red binding to begin with and showed an increase by about 15% of lipid content, which was not significant, though not surprising, as the cells were derived from individual isolates.

**Figure 3:**
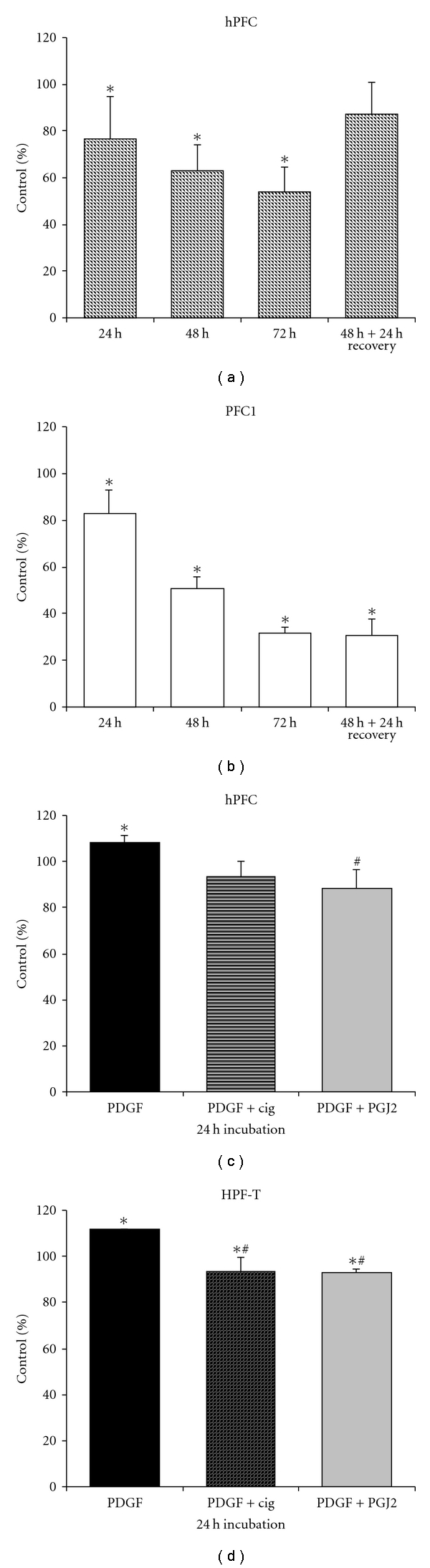
Effects of PPAR*γ* ligands on proliferation. (a) Time course of incubation of hPFC with 3.3 *μ*M ciglitazone. Proliferation decreases significantly (*) over time. Reversibility was tested by washout of drug after 48 h and incubation in fresh drug-free medium for further 24 h, and the proliferation rate did not significantly differ from untreated cells. (b) Time course of incubation of PSC-1 cells with 3.3 *μ*M ciglitazone. Proliferation rates decreased significantly (*) over time. The washout experiment did not show reversibility of ciglitazone-induced inhibition. (c) Effect of PPAR*γ* ligands on PDGF-stimulated proliferation in hPFC. Cells were incubated for 24 h with platelet-derived growth factor (PDGF) at a concentration of 5 ng/mL. One hour after the start of PDGF incubation, ciglitazone (cig, 3.3 *μ*M) or 15Δ-prostaglandin J_2_ (PGJ2, 5 *μ*M) was added. PDGF induced a significant growth stimulation. Ciglitazone treatment did not significantly inhibit this growth induction, but PGJ2 did. (*Significant versus control, ^#^significant versus PDGF stimulation.) (d) Effect of PPAR*γ* ligands on PDGF-stimulated proliferation in HPF-T cells. Cells were incubated for 24 h with platelet-derived growth factor (PDGF) at a concentration of 5 ng/mL. One hour after start of PDGF incubation, ciglitazone (cig, 3.3 *μ*M) or 15Δ-prostaglandin J_2_ (PGJ2, 5 *μ*M) was added. PDGF stimulated proliferation significantly, and both ciglitazone and 15Δ-prostaglandin J_2_ inhibited stimulated growth in transformed HPF-T cells. (*Significant versus control, ^#^significant versus PDGF stimulation, *n* ≥ 3.)

We then tested for effects of PPAR*γ* ligands on cell proliferation, using a tetrazolium blue-based assay that reflects mitochondrial activity, which is proportional to cell numbers.  Figure 3 shows results for different treatments on PFC1, hPFC, and HPC-T cells. Cells were incubated with 3.3 *μ*M ciglitazone for 24 to 72 h, and hPFC showed a significant decrease in proliferation (Figure 3(a)). To test for reversibility of this effect, whether it was due to growth inhibition or cell death, cells were treated for 48 h with ciglitazone and then washed and incubated without the drug. After 24 h of chase, cell proliferation increased to numbers not significantly different from controls. Spontaneously immortalized rat cells (PFC1, Figure 3(b)) incubated with 3.3 *μ*M ciglitazone also showed significant reduction of proliferation but did not recover from 48 h ciglitazone treatment after 24 h chase. Further tests were performed on the effect of PPAR*γ* ligands on PDGF- (platelet-derived growth factor)-induced proliferation (Figures 3(c) and 3(d)). We tested not only for the synthetic ligand ciglitazone, but also the natural ligand 15-Δ-prostaglandin J_2_ (PGJ2). Treatment of hPFC with 5 ng/mL PDGF for 24 h resulted in significant stimulation of proliferation, and incubation of PDGF- stimulated cells with ciglitazone reduced the stimulating effect. The reduction of PDGF-stimulated proliferation was significantly more pronounced with the natural ligand 15-Δ-prostaglandin J_2_ (Figure 3(c)). For the transformed cell line HPF-T, the results were similar, with significant reduction of PDGF-stimulated proliferation by synthetic and natural PPAR*γ* ligands (Figure 3(d)).

**Figure 4:**
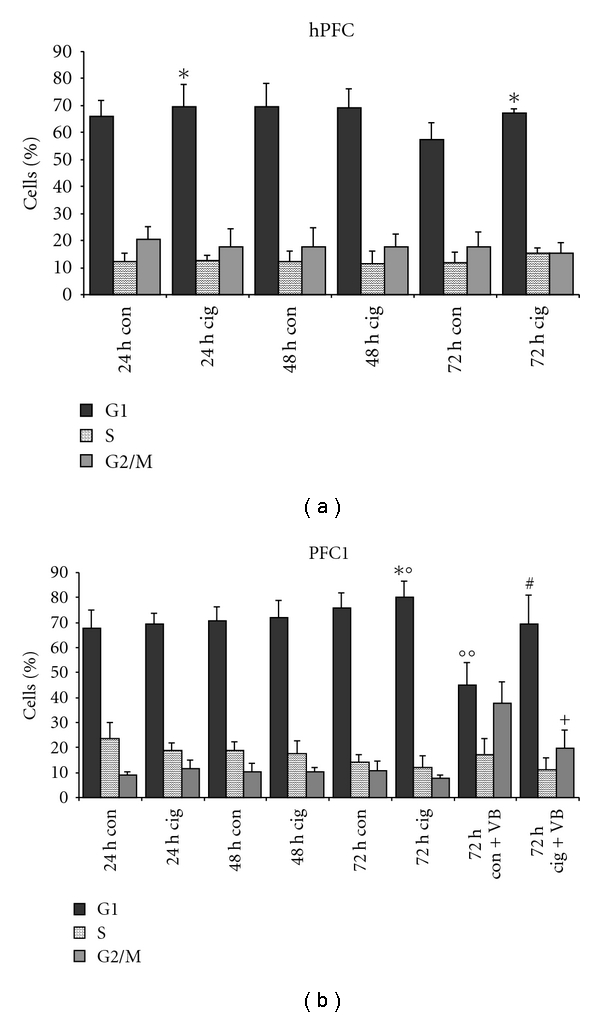
Cell cycle analysis. (a) Cell cycle analysis in hPFC cells after 24 h, 48, and 72 h of treatment with 3.3 *μ*M ciglitazone. The rise in G1 phase cells (black) was significant after 24 h (*) and 72 h (*) compared to concomitant control values. S phase is depicted in hatched columns, and G2/M phase is depicted in grey, *n* ≥ 3. (b) Cell cycle analysis in PFC1 cells shows a steady rise in G1 phase cells over the time of culture, no matter if treated or not. At 72 h of ciglitazone treatment, the difference compared to controls was significant (*), as well as compared to 48 h of treatment (*n* ≥ 3). Vinblastine- (VB-) treatment showed a significantly reduced number of G1 cells compared to 72 h untreated controls (°°) and 72 h ciglitazone treatment (°), and a high level of G2/M phase cells. These effects were diminished by ciglitazone: G1 phase cell numbers were not significantly different from 72 h ciglitazone only (^#^), and G2/M phase accumulation was significantly reduced (^+^).

For further investigation of the antiproliferative effect of ciglitazone, cell cycle analysis was performed over the time course of 24 to 72 hours at 3.3 *μ*M concentration, as depicted in Figure 4. Propidium iodide staining for DNA content showed a significant increase of G1 phase cells after 24 h and 72 h of treatment in hPFC (Figure 4(a)). In transformed HPF-T cells, we found a higher rate of apoptosis during standard culture, which was significantly enhanced by ciglitazone treatment after 48 h and 72 h (data not shown). PFC1 cells were tested as well (Figure 4(b)) and showed a constant rise in G1 phase cells from 24 h to 72 h in control cells and after ciglitazone treatment. As the increase in cell numbers in G1 phase was moderate, vinblastine was employed [18]. Vinblastine induces a cell cycle arrest in G2/M phase. After 72 h of vinblastine treatment, G1 phase cells were significantly reduced while cells accumulated in G2/M phase. This effect was inhibited by ciglitazone treatment. The percentage of cells in G1 phase under combined treatment with ciglitazone and vinblastine was not significantly lower than after ciglitazone only treatment for 72 h. Furthermore, cell numbers in G2/M phase were significantly lower under the combined influence of vinblastine and ciglitazone than when treated with vinblastine only. So the effect of ciglitazone on cell cycle progression was confirmed. 

**Figure 5:**
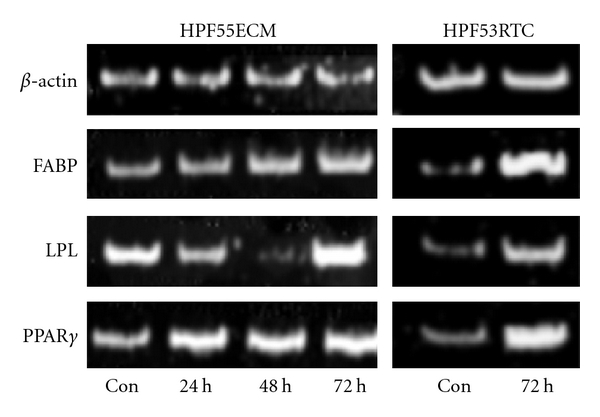
PCR Analysis of lipid metabolism proteins. PCR analysis was carried out on two individual isolates of hPFC cells under control conditions (con) and after 24 h, 48 h, and 72 h ciglitazone treatment at 3.3 *μ*M ciglitazone for HPF55ECM cells, and control and 72 h ciglitazone at 3.3 *μ*M for HPF53RTC cells. For normalisation, *β*-actin was amplified. Fatty acid binding protein (FABP) showed a steady rise in expression over 24–72 h being highest at 72 h. Lipoprotein lipase was induced after 72 h of ciglitazone treatment though mRNA levels showed high variations, even under control conditions between isolates. PPAR*γ* was induced at mRNA level after 24 h of treatment and highest after 72 h for both samples tested. PCR analysis shows the differences between individual isolates of hPFC, while the general responsiveness towards ciglitazone treatment was similar, basal expression and induction values varied.

In order to confirm the influence of ciglitazone on hPFC, we investigated the gene regulation of proteins involved in lipid metabolism.  Figure 5 shows RT-PCR analysis of fatty acid binding protein (FABP), lipoprotein lipase (LPL), and PPAR*γ* itself in two individual isolates of hPFC. Over the time course of 24 to 72 h, RNA levels of FABP rose continually, while LPL varied in RNA levels, with a maximum after 72 h. PPAR*γ* itself was induced after 24 h of ciglitazone treatment and maintained the higher levels. These findings were confirmed, when another isolate was investigated after 72 h of ciglitazone treatment. Human samples were derived from patients with pancreatic cancer. Specimens were taken from morphologically normal areas of tissue after tumour resection. All analyses on lipid content and induction of lipid metabolism were carried out on cells of the same original isolates.

**Figure 6:**
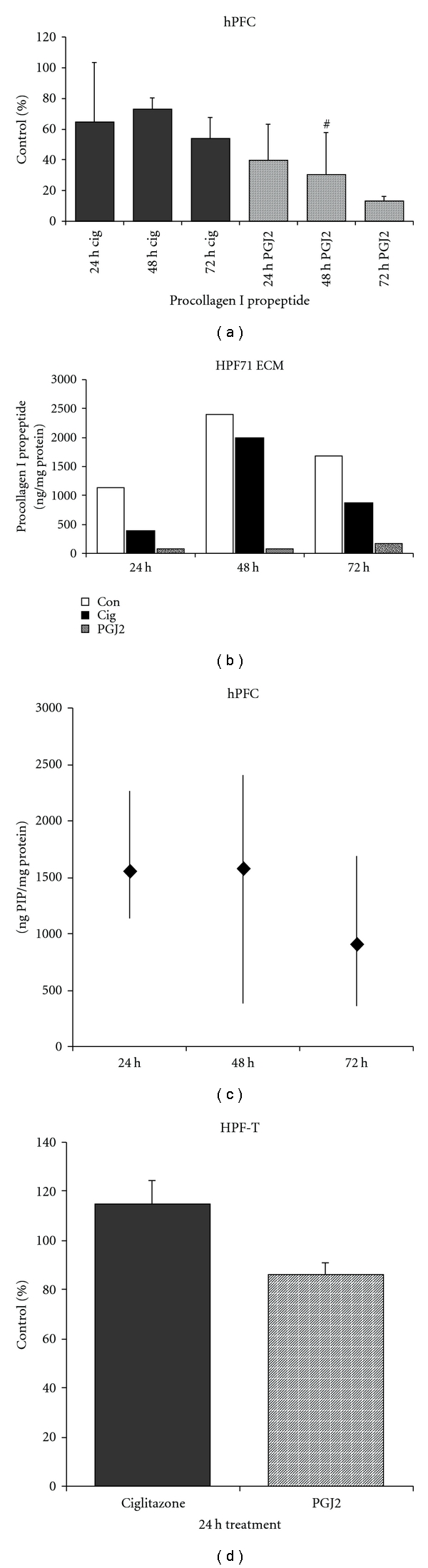
Analysis of collagen synthesis. (a) Procollagen I propeptide (PIP) secretion of hPFC presented as percent of control after 24, 48, and 72 h of ciglitazone (3.3 *μ*M) and PGJ2 (5.0 *μ*M) treatment. The reduction of PIP content in culture supernatants was significant, except for 48 h PGJ2 (#, *n* ≥ 3). (b) An example of PIP as ng/mg protein for an individual isolate, representing an individual response towards treatment, and changes in cellular collagen synthesis over time in culture. (c) Depiction of collagen synthesis in control cells of the individual isolates used in this analysis as mean, minimum, and maximum values (*n* ≥ 3). (d) Collagen synthesis in transformed cells (HPF-T) after 24 h of 3.3 *μ*M ciglitazone and 5.0 *μ*M PGJ2 treatment expressed as percent of control. Within the first 24 h, HPF-T cells responded to ciglitazone by a significant induction of collagen synthesis, while the natural ligand inhibited collagen synthesis significantly (*n* ≥ 3).

We further tested for the effect of PPAR*γ* ligands on collagen synthesis, using an immunoassay for procollagen I propeptide. During collagen fiber assembly, the propeptide is cleaved and concomitantly released into the cell culture supernatant and can be assessed by immunological techniques. The assay used was specific for human procollagen I C peptide.  Figure 6(a) shows that in hPFCs ciglitazone and the natural ligand 15Δ-prostaglandin J_2_ inhibit collagen synthesis after 24, 48 h, and 72 h of incubation, expressed as percent of controls. Apparently, the natural ligand is more effective than ciglitazone in inhibition of collagen synthesis. As seen before, this analysis revealed that collagen synthesis in individual hPFC isolates showed differences; furthermore, synthesis varied throughout the investigated time course.  Figure 6(b) gives an example of collagen synthesis and inhibition by PPAR*γ* ligands for one individual isolate named HPF71ECM, from a patient treated for pancreatic cancer.  Figure 6(c) shows the range of collagen synthesis for all untreated controls of hPFC isolates tested at different times of incubation; in general, collagen synthesis appeared to be maximal 2 d after plating and decreased with ongoing culture, while the amount of collagen synthesis differed.  Figure 6(d) shows results for the transformed human cell line HPF-T, expressed as percent of control. Ciglitazone treatment for 24 h significantly induced collagen synthesis, while PGJ2 treatment resulted in significant inhibition. Prolonged treatment with ciglitazone led to inhibition of collagen synthesis (data not shown). 

**Figure 7:**
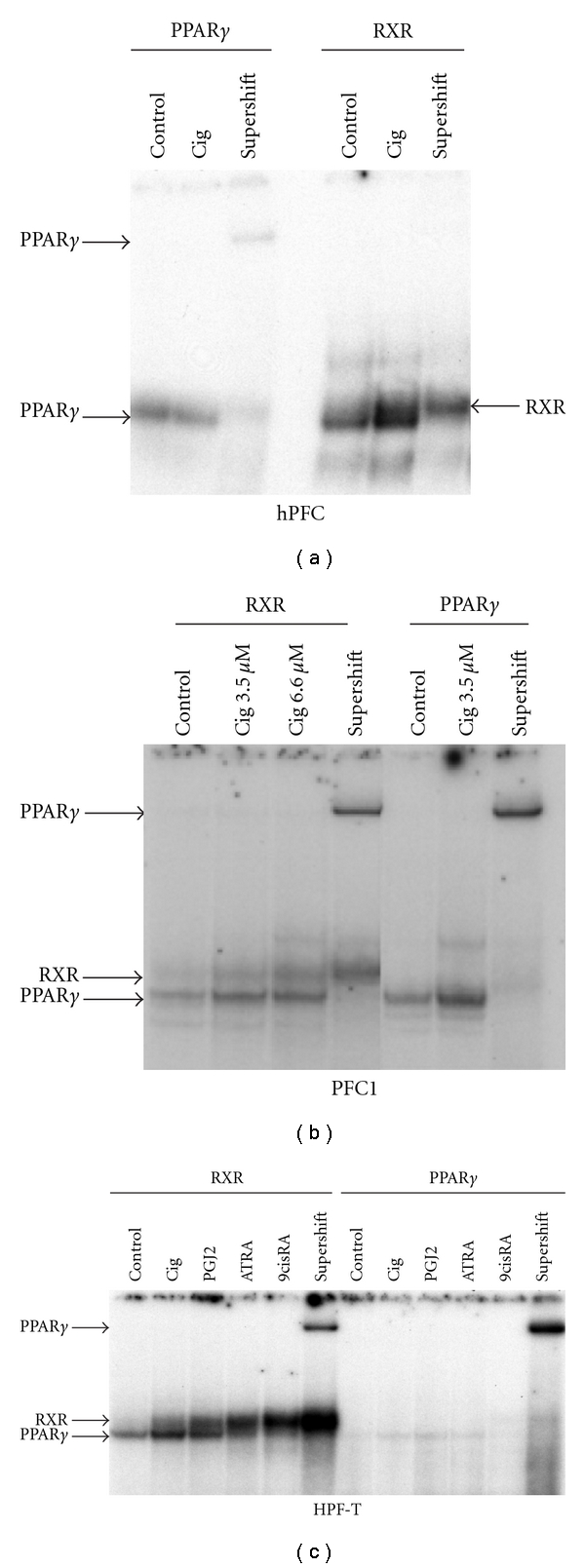
Gel shift analysis of PPAR*γ*. (a) Electrophoretic mobility shift analyses (EMSAs) in hPFC showed DNA binding of PPAR*γ* under control conditions, in the absence of ligand and in response to ciglitazone (3.3 *μ*M) stimulation using a PPAR*γ* consensus sequence. Supershift analyses with anti-PPAR*γ* antibodies were included to show the specificity of PPAR*γ* binding. Binding to a retinoid X receptor (RXR) consensus sequence was visible without ciglitazone treatment, but more pronounced after ligand binding to PPAR*γ*. Supershift analysis revealed that PPAR*γ* was present in the complex binding to RXR. (b) Analysis of PPAR*γ* binding in rat PFC1 cells. Two different concentrations of ciglitazone were used to show induction of dimer formation and binding to the RXR consensus sequence. A clear induction is visible with the PPAR*γ* probe already at the lower concentration. Supershift analysis showed that principally the lower of the two bands contained PPAR*γ*, while the upper band remained in place. (c) Analysis of PPAR*γ* binding in transformed HPF-T cells. Different stimulators were used for differentiation purposes. Analysis of stimulation with ciglitazone (3.3 *μ*M) was compared to 15Δ-prostaglandin J_2_ (PGJ2 at 5 *μ*M), next to all trans retinoic acid (ATRA) and 9-cis-retinoic acid (9cisRA). The lower PPAR*γ* band was induced probing with RXR by ciglitazone and PGJ2, but ATRA and 9cisRA inductors for retinoid receptors did not stimulate PPAR*γ* binding to this consensus sequence. Using the consensus sequence of rat acyl coenzyme A oxidase, the signal was considerably weaker, but still induction of receptor binding was induced by specific ligands, while RXR binding to this probe appeared reduced.

PPAR*γ* action in gene regulation was further investigated by DNA binding studies, that is, electrophoretic mobility shift assays (EMSAs), to confirm ciglitazone-induced activity in the cells investigated.  Figure 7 shows binding of PPAR*γ* to different consensus sequences for the three cell types investigated in this study. PPAR*γ* binds to DNA as a heterodimer with retinoid X receptor (RXR), thus we used probes with RXR binding sites as well as PPAR*γ* consensus sequences and performed supershift analyses with antibodies specific for PPAR*γ* to confirm the presence of the molecule in the heterodimers. Analysis of an individual hPFC isolate revealed that PPAR*γ* bound in the absence of ligand, which is in accord with a corepressor binding in the absence of PPAR*γ* ligands, which is released upon ligand binding to the dimer [19]. Incubation with 3.3 *μ*M ciglitazone for 1 h induced PPAR*γ* binding to the RXR consensus sequence (Figure 7(a)). For PFC1 cells, we showed a dose-dependent increase in PPAR*γ* binding to both probes investigated (Figure 7(b)). In HPF-T cells, we analysed the effects of different inductors for PPAR*γ* and RXR. Ciglitazone as well as the natural ligand PGJ2 induced PPAR*γ*, while RXR binding was enhanced by retinoic acid-derivatives like all-trans retinoic acid (ATRA) and 9-cis-retinoic acid (9cisRA), the former inducing preferentially retinoic acid receptor (RAR), while the latter has been shown to induce retinoid X receptor (Figure 7(c)). Pronounced binding was found for the RXR consensus sequence after PPAR*γ* ligand stimulation. Stimulation with 9cisRA appeared more efficient than ATRA. The PPAR*γ* consensus sequence showed considerably less binding but still revealed similar outcomes.

## 4. Discussion

Isolation procedures for pancreatic cells of fibroblast lineage or characteristics yield different cell populations. Pancreatic stellate cells, mostly isolated from rat pancreas by tissue digestion and differential centrifugation [1, 2], produce cells which show lipid storage, as detected by oil red staining; these cells are isolated by their buoyant density. Loss of lipid content would be detrimental to the isolation procedure. On the other hand, cells isolated by outgrowth from tissue pieces in cell culture are per se activated, they must migrate to leave their environment, and they undergo cell division in cell culture prior to being harvested as a passageable cell population with features of fibroblasts [12]. In this study, we investigated human pancreatic fibroblast-like cells isolated by outgrowth, and one of the aims was to analyse whether these cells have the capability of lipid storage. Ultrastructural analysis revealed that at the earliest time point of investigation, there were essentially no lipid droplets resembling those found in rat pancreatic stellate cells by size or number. However, there appeared to be a considerable amount of stacked lipid bilayers concentrated in membrane-bounded vesicles, which might give rise to smaller, secondary lipid storing structures with a distinct layering and without a containing lipid bilayer membrane. As structures like these are barely seen in tumour cells or others of epithelial origin, the potential for lipid storage appears to be inherent in mesenchymal/fibroblast lineages. It is generally believed that the activation process for stellate cells goes along with loss of lipid content and expression of alpha smooth muscle actin as a marker. We show here that isolation of a priori activated cells from different individuals with different etiology of disease, leading ultimately to surgical intervention, yields cells which are heterogeneous in appearance but do respond to pharmaceutical manipulation in a coordinated manner. Thiazolidinediones, used as antidiabetics, were shown to inhibit proliferation and activation of rat PSC [6] and immortalized rat PSC [10] in vitro. Similar results were found for hepatic stellate cells (HSCs) in vitro [20]; most of these experiments were carried out on cells for rat origin. Furthermore animal models for chronic pancreatitis [7] and liver fibrosis [21] did respond to treatment with TZDs, PPAR*γ* ligands by ameliorated fibrotic responses, though there is also a report on a mouse model of hepatic fibrosis that was not responsive towards pioglitazone treatment [22]. So far, little is known about human pancreatic fibroblasts in vitro. Investigation of PPAR gene expression in human tissues [23] revealed a maximum of PPAR*γ* 1 and 2 expression in human fat tissue, and to a lower extent in liver, heart, and muscle. Rats and mice show a similar prevalence in PPAR*γ* 2 expression in fat tissue, but expression levels are far lower than in human tissues, which is even more evident for PPAR*γ* 1. We found PPAR*γ* mRNA in hPFC untreated control cells after prolonged time in culture, readily induced by ciglitazone after 24 h, and in rat HSCs, PPAR*γ* expression went down over time in culture but was inducible by PGJ2 [24]. However, the most successful model for induction of adipocyte differentiation is MDI (isobutylmethylxanthine/dexamethasone/insulin) treatment, which usually lasts for 3 days followed by a more or less extended culture period which was initially developed for mouse 3T3 L1 cells ([22] and references therein) and was successfully applied to rat HSC [25]. In HSC, MDI treatment restored a quiescent phenotype by induction of genes regulating lipid metabolism, an effect also seen by overexpression of PPAR*γ* [26]. We report here the induction of FABP and LPL as representatives of lipid metabolism, as well as PPAR*γ* mRNA induction after ciglitazone treatment. Furthermore, we report on DNA binding in heterodimers with retinoid X receptor, thus ensuring direct involvement of PPAR*γ* in enhanced lipid content.

However, 72 h of treatment might not be sufficient for a full-fledged adipogenic differentiation in human or rat pancreatic fibroblastoid cells but clearly points towards activation of signalling pathways leading to reduced proliferation and enhanced lipid content, similar to induction of adipogenic differentiation. In addition, we were able to show a reduction of collagen synthesis by PPAR*γ* ligands, arguing for reversibility of the activation process. It is tempting, to speculate, that a reversal of activation by synthetic PPAR*γ* ligands might work in humans by promoting the inherent adipogenic potential of pancreatic interstitial fibroblasts.
